# Ventricular bigeminy and trigeminy caused by hypophosphataemia during diabetic ketoacidosis treatment: a case report

**DOI:** 10.1186/s13052-019-0633-y

**Published:** 2019-04-02

**Authors:** Konrad Miszczuk, Joanna Mroczek-Wacinska, Robert Piekarski, Beata Wysocka- Lukasik, Renata Jawniak, Iwona Ben-Skowronek

**Affiliations:** 1 Department of Pediatric Endocrinology and Diabetology, Medical University of Lublin, Lublin, Poland; 2 Department of Pediatric Cardiology, Medical University of Lublin, Lublin, Poland

**Keywords:** Diabetic ketoacidosis, Hypophosphatemia, Ventricular arrhythmia, Case report

## Abstract

**Background:**

Hypophosphatemia has many causes, and is often encountered during DKA (Diabetic Ketoacidosis) treatment. However, it rarely requires clinical intervention.

**Case presentation:**

Ventricular arrhythmia was observed in a 10-year-old girl with newly diagnosed type 1 diabetes mellitus and hypophosphatemia while undergoing treatment for ketoacidosis. Oral phosphate supplementation ceased ventricular arrhythmia almost completely.

**Conclusions:**

The clinical signs of hypophosphatemia are potentially life-threatening. Therefore, physicians should be vigilant when treating patients who are at risk of hypophosphatemia. Severe hypophosphatemia accompanied by clinical symptoms requires oral or intravenous supplementation of phosphate.

## Background

Diabetic ketoacidosis (DKA) is a life-threatening condition that requires immediate treatment. Type 1 diabetes mellitus is an autoimmune disease, in which beta-cell destruction process starts as cellular reaction that leads to beta-cell damage that stimulates autoimmune humoral reaction. As the level of anti-pancreatic autoantibodies rises, insulin secretion is impaired leading to complete lack of insulin. As a result, hyperglycemia occurs and leads to ketoacidosis. The initial symptoms such as polydipsia, polyuria and nycturia lead to dehydration. As acidosis progresses, the patient presents elevated heart rate, elevated breathing rate and Kussmaul breathing pattern occurs. These symptoms are often accompanied by stomach ache, nausea and vomiting. As condition worsens, neurological symptoms, ranging from confusion and sleepiness to coma, may occur. The laboratory criteria for DKA diagnosis are: Hyperglycemia (plasma glucose > 200 mg/dL), serum pH < 7,3 or serum bicarbonate level < 15 mmol/L and also the presence of serum ketones and ketonuria. DKA severity is determined by the level of acidosis: in mild DKA pH is 7,2–7,3 or bicarbonate level is 10–15 mmol/L; in moderate DKA pH is 7,1–7,2 or bicarbonate level is 5–10 mmol/L; in severe DKA pH < 7,1 or bicarbonate level < 5 mmol/L. Essential treatment requires fluid resuscitation and human recombinant insulin administartion in continuous IV infusion. Patient overall condition should be monitored continuously, blood glucose and neurological status should be checked every hour, serum electrolytes should be checked every 2 h after starting IV fluids. Additionally ECG should be monitored for abnormal T waves and T waves changes. Cerebral edema and hypokalemia are the most common complications during DK treatment. Other possible complications include severe hypophosphatemia, hypocalcaemia, hypomagnesaemia, hypoglycemia, heart arrhythmias, deep vein thrombosis, pulmonary embolism, sepsis, pulmonary edema, acute respiratory distress syndrome, rhabdomyolisis, ischemic renal necrosis, acute renal failure, acute pancreatitis [1].

Phosphorus is an element which is essential for life. The human body contains 11 to 14 g/kg of lean body mass [2]. The majority of phosphorus is concentrated in the bones (600–700 g), and is also found in the soft tissue (100–200 g). Apart from its structural function, it plays a crucial role in cellular metabolism and in cell membranes. Phosphorus is a part of adenosine triphosphate (ATP), phosphocreatine and 2,3-diphosphoglycerate (2,3-DPG) [3].

Food is a rich source of phosphorus, especially dairy products, meat and beans. It is estimated that 1 ml of milk contains 1 mg of phosphorus. A typical diet provides 800–1400 ng of phosphorus daily, and approximately 65% of ingested phosphorus is absorbed in the intestines [4, 5].

The normal serum concentration of phosphate fluctuates with age, with the normal concentration for adults ranging between 0.8 and 1.3 mmol/L. Mild hypophosphatemia is defined as a phosphate level between 0.32 and 0.65 mmol/L, while severe hypophosphatemia is diagnosed when the phosphate level drops below 0.32 mmol/L [6].

A shift in phosphate level is expected to occur in DKA, resulting both from the pathogenesis of the disease and insulin action. Although changes in serum phosphate level are widely described [7, 8], there is still need for studies on large research group in that matter. T.Shen and S.Braude evaluated 64 patients with DKA. 63% of the patients had hyperphosphatemia on admission, 33% of the patients had phosphate level within the normal range and 5% of the patients suffered from hypophosphatemia. The phosphate level decreased during DKA treatment in all cases. In 90% nadir was < 0,8 mmol/L, in 36% nadir was < 0,5 mmol/L and severe hypophosphatemia (phosphate level below 0,32 mmol/L) developed in 11% of the cases [9].

There are multiple issues related to the interpretation of serum inorganic phosphorus (Pi) levels. Firstly, there is no simple translation of serum phosphate levels to the severity of clinical signs [6]. Secondly, there is diurnal variation in serum phosphate levels. Finally, hypophosphatemia does not necessarily reflect a deficit in total body phosphorus content, as the intracellular content is 100-times higher than the serum level. It is important to take into consideration that the clinical condition of the patient depends on the intracellular concentration of phosphate [6]. Clinically symptomatic hypophosphatemia is more likely to occur in patients who are nil by mouth, those with impaired food absorption, patients with abnormal intracellular-extracellular phosphate distribution, and patients who lose phosphates via urinary excretion [2, 4] (Table 1).

**Table 1:** Causes of hypophosphatemia

Insufficient inorganic phosphate delivery with food	
- Premature birth	
- Anorexia nervosa	
- Parenteral feeding with a low-phosphorus diet	
- Drug-binding phosphates	
- Alcohol addiction	
An abnormal shift of inorganic phosphate (Pi) from the extracellular space to the intracellular space	
- Insulin and glucose infusion	
- Refeeding syndrome	
- Hungry bones syndrome	
- Complete parenteral feeding	
- Anabolic state in patient with severe burn or trauma	
- Respiratory alkalosis	
- Tumor progression	
- Bone marrow transplant	
Excessive urinary phosphate loss	
- Hypophosphatemic rickets	
- Tumor-induced rickets	
- Fanconi syndrome	
- Vitamin D deficiency	
- Glucocorticosteroid overdose tubular acidosis	
- PTH (parathormone) excess	
- Presence of PTHrP (Parathyroid hormone-related protein)	

The life-threatening clinical manifestations of hypophosphatemia are associated with the energetic role of phosphate. As a part of ATP, phosphate is a key element in cell energy turnover. Furthermore, 2,3-DPG deficiency in red blood cells promotes oxygen binding to hemoglobin, therefore oxygen is less likely to be released to the tissues that need it most. Ischemia occurs as a result, which affects both tissues and organs.

The main clinical manifestations of hypophosphatemia include metabolic encephalopathy (including signs of irritability, paraesthesia, seizures, coma), impaired cardiac contractility (including cardiomyopathy), heart arrhythmia (ranging from atrial fibrillation to PVC - Premature Ventricular Contractions), respiratory failure, impaired skeletal muscle contractility (starting from proximal to distal), rhabdomyolysis, dysphagia and hemolysis [1, 2, 4, 10–14].

## Case report

A 10-year-old girl was admitted to our clinic due to ketoacidosis associated with newly diagnosed type 1 diabetes. Prior to admission, she had suffered from polyuria, polydipsia, and nycturia for about 2 weeks, and presented with fatigue, drowsiness and a lack of appetite for the last two days. Her history did not include any chronic diseases, and she had normal growth and development.

On admission, she was in a serious state, suffering from vomiting and dehydration, and displaying Kussmaul breathing, a respiratory rate of 45 breaths/min and tachycardia. The blood test revealed a blood glucose level of 26.8 mmol/L (482 mg/dl), pH 6.902, base excess (BE) of − 29.3 mmol/L, HbA1c of 12.9%, sodium level of 142 mEq/L, potassium level of 4.11 mEq/L and ketone level of 6.1 mmol/L. The patient was treated according to the recommendations of the International Society of Pediatric and Adolescent Diabetes and the Polish Diabetes Association [1, 15].

Initially the patient received 500 ml of 0.9% NaCl IV infusion. Next, a continuous IV infusion of short acting insulin was commenced with 2,5 U/ h dose, taking into consideration that the patient weight was 40 kg. IV Insulin infusion was sustained for 51 h with dosages ranging from 1,5 U/h to 4 U/h. Overall the patient received 81,75 units of IV insulin during the first 24 h of treatment. Moreover, potassium was supplemented intravenously accordingly to changes in electrolytes results. Although she remained in a serious condition during the first 24 h of treatment, she stopped vomiting and showed gradual improvement in blood test results, with pH normalization and a decrease in the level of ketones. The glucose level was maintained between 8.3 and 13.9 mmol/L (150–250 mg/dl) with intravenous insulin and glucose infusions. The neurological status was checked hourly, and was reported as stable. An ECG was performed within the first few hours of admission, which did not reveal any crucial abnormalities aside from tachycardia. The patient received 65 units of IV insulin during the second 24 h f treatment. During the whole continuous insulin infusion the patient fluid input was 7850 ml (4750 ml IV, 3100 ml orally) and diuresis was 3400 ml.

**Fig. 1:**
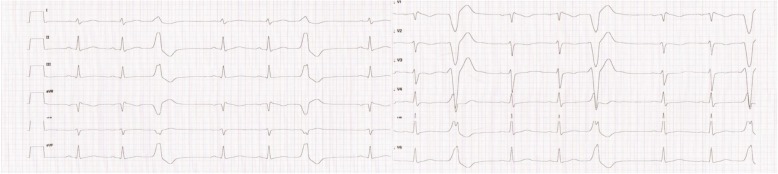
An electrocardiogram with premature ventricular complexes performed after the first recognition of the irregular hearth rhytm

The patient’s overall condition improved during the second day of treatment, despite the fact that she was still receiving intravenous insulin. That day irregular heartbeats were noticed on auscultation during the physical examination and on the ECG monitor. A second ECG showed premature ventricular complexes (PVCs; also known as trigeminy) with LBBB (left bundle branch block) morphology and a normal axis (Fig. 1). Continuous ECG monitoring was subsequently implemented, and the patient underwent a cardiac consultation. The echocardiogram showed no abnormalities in the structures of the heart and great vessels. The consultant cardiologist confirmed the diagnosis of ventricular arrhythmia (PVCs). The blood results revealed hypokalemia (nadir at 2.9 mmol/L) and hypophosphatemia (nadir at 0.45 mmol/L).

The ECG performed after the first recognition of the patient’s the irregular heart rhythm (Fig. 1) showed: sinus rhythm at 100/min, normal cardiac axis, with premature ventricular complexes. Normal PQ interval (0,14 s), normal QRS duration (0,08 s), normal QT interval (0,31 s)and QTc 0,40 QTd 0,04 s. Normal T wave and repolarization. The biochemistry results at that moment were: Na 134 mmol/L Na 134 mmol/l [132–145]; K 3,56 mmol/l [4,1-5,3]; Ca 2,70 mmol/l [2,1-2,6]; Mg 0,83 mmol/l [0,53-1,11]; Phosphate 0,45 mmol/l [1,1-2,0] (assessed for the first time); pH 7,34 [7,35-7,45]; BE-11,5 mmol/l [− 2,0/+ 3,0]; HCO3 14,2 mmol/l [22,5-30,0]. CK level was not determined. The pattern visible in the ECG was not typical of hypokalemia, which is connected with initial T-wave decrease, followed by ST-segment depression, T-wave inversion, PR interval prolongation and increased P-wave amplitude, observed with further decreases in serum potassium levels. Severe hypokalemia may be associated with giant U-waves created by T- and U-wave fusion. Tachyarrhythmia and rarely atrioventricular block may evolve [16–18]. In our case we did not observe such abnormalities typical to low potassium level in the ECG. Moreover, the ECG was not typical of hypercalcaemia, which causes prolonged PQ interval and shortening of QT interval [19, 20]. Taking above into consideration we propose possible association between hypophosphatemia and premature ventricular complexes reported in the ECG.

Potassium was supplemented both orally and intravenously. Overall the patient received 123 mEq of potassium during the continuous insulin therapy. As far as phosphorus is concerned, the patient was fed with liquid dairy products that were rich in phosphate, as she was only able to swallow small amounts of non-solid foods due to odynophagia. She was also started on oral phosphate supplementation (phosphate mixture containing 17.8 g Na_2_HPO_4_ and 4.88 g NaH_2_PO_4_ in 100 ml of distilled water, containing 1.6 mmol of inorganic phosphate per 1 ml). As 1 mmol of inorganic phosphate weighs 31 mg, our patient was receiving 62 mg Pi/kg body weight every 24 h from the phosphate mixture. She continued to present an irregular heart rate, thus a 24-h ECG Holter monitor examination was performed. The results showed 38,000 monomorphic PVCs, 1615 occurrences of ventricular bigeminy, 1230 of ventricular trigeminy and 453 of ventricular quadrigeminy, which comprised 38% of all the heart electrical activity recorded.

The patient was started on intensive subcutaneous insulin regimen at the 3 day of treatment, after the biochemistry results improved. In the beginning she was receiving 15 units of ultra- long acting basal insulin and 27,5 units of rapid acting insulin for meals and correction doses.

The patient’s serum phosphate level normalized over the few next days of treatment (Table 2), and ventricular arrhythmia ceased almost completely. Subsequent ECG Holter examination showed 1760 PVCs, 13 episodes of ventricular bigeminy and 79 episodes of ventricular trigeminy. Additionally, we noticed hypocalcaemia after 3 days of phosphate supplementation. Taking into consideration the patient’s severe vitamin D3 deficiency (25(OH)D3 level 9.83 ng/ml), we supplemented both calcium and vitamin D3.

**Table 2:** Serum electrolyte levels during treatment with phosphate formulation

Day of treatment	Day 1	Day 2	Day 3	Day 5	Day 6	Day 7	Day 8	Normal range
Natrium (mmol/L)	142	134	142	139	144	144	141	132–145
Potassium (mmol/L)	4.11	3.56	4.02	2.92	3.15	3.89	4.29	4.1–5.3
Calcium (mmol/L)	2.10	**2.70**	2.42	2.20	**1.83**	**1.98**	2.35	2.1–2.6
Phosphate (mmol/L)	–	**0.45**	**1.42**	**0.90**	0.97	1.32	1.19	1.1–2.0
Phosphate formulation (10 ml orally five times a day)	–	Beginning of drug administration	Break	+	+	+	Termination of drug administration	

In bold abnormal concentrations of calcium and phosphate

Gradually the patient regained insulin sensivity and entered a partial remission phase. C- peptide level was 0,84 ng/ml.

The patient was discharged in an overall good condition receiving intensive functional insulin therapy. The regimen included 10 units of basal insulin administered once daily in the evening and rapid acting insulin with insulin: carbohydrate ratio set for each meal and also with recommended correction doses.

A cardiac check-up at the outpatient clinic following discharge revealed no signs of cardiac arrhythmia. The patient is currently on an insulin pump therapy regimen, and her growth and development are within the normal range.

## Discussion

The case presented here involved multicausal hypophosphatemia. Diabetic ketoacidosis led to a loss in body phosphorus content. This occurred firstly due to osmotic diuresis, resulting in urinary phosphate loss [9]. Secondly, insulin therapy is known to directly cause a reduction in serum phosphate levels [21]. In addition, variations in the acid-base balance also contribute to hypophosphatemia as, when the pH increases, an intracellular shift of phosphate from the serum takes place [22]. In our patient, vitamin D3 deficiency was another factor that contributed to the low total body phosphate stores [23]. Moreover, the loss of appetite caused a significant decrease in oral phosphate intake. As the patient began eating on the second day of treatment, refeeding syndrome had to be taken into consideration. Taken together, each of these factors contributed to the occurrence of ventricular arrhythmia, despite the serum phosphate level of 0.46 mmol/L not meeting the criteria for severe hypophosphatemia.

We believe that the clinical manifestations of hypophosphatemia were caused not only by the serum phosphate level, but also by a low intracellular phosphate content.

Heart arrhythmias are rare complication of DKA so there is lack of large-group research on that topic. The data available depicts case reports with supraventricular tachycardia (SVT), ventricular tachycardia and atrial fibrillation, which authors describe as multi etiological [24–29].

Arrhythmia associated with hypophosphatemia are mainly supraventricular arrhythmia, and may also include single preliminary ventricular complexes, ventricular bigeminy and trigeminy [30]. Ventricular extrasystoles are considered an early marker of myocardial ischemia [30, 31]. In our case, ventricular arrhythmia ceased almost completely after oral phosphate supplementation and normalization of serum phosphate levels.

Many authors [1, 2, 5, 15] have reported that administration of phosphates in DKA does not improve prognosis, and indeed increases the risk of hypocalcaemia. In our case, hypocalcaemia occurred on the fifth day of treatment, and the patient was treated with calcium supplementation. Routine administration of phosphates in DKA is not recommended. The only indication for the use of phosphates is severe hypophosphatemia (phosphate level of < 0.32 mmol/L) or the presence of clinical symptoms of hypophosphatemia such as ventricular arrhythmias [1].

Phosphate may be administered orally or intravenously, depending on the patient’s condition (Table 3). Oral administration is preferred as it is recognised to be safer and more effective. However, it is difficult to anticipate the dose effect, as serum phosphate levels do not reflect total body phosphate stores. Calcium, phosphate, magnesium, potassium and creatinine levels need to be closely monitored during phosphate supplementation. ECG should be performed every 12–24 h during oral therapy, and up to every 6 h during intravenous therapy. Patients with renal failure are prone to developing hypophosphatemia.

**Table 3:** Phosphate and vitamin D supplementation in severe acute hypophosphatemia

Oral phosphate salts at a dose of 30–40 mg/kg body weight per day, administered in 4–5 doses.	
Cholecalciferol (vitamin D3) at a dose of 800–1000 U/day (increase the dose in case of deficiency).	
Intravenous phosphate solution at the following doses:	
- 0.08 mmol/kg body weight for 6 h, recommended for short-term hypophosphatemia without complications;	
- 0.16 mmol/kg body weight for long-term hypophosphatemia; or	
- In some cases, doses up to 0.4–0.5 mmol/kg body weight for 6 h are required, up to a maximum dose of 50 mmol of phosphate.	

The recommended oral dosage of phosphate is 30–40 mg Pi/kg of body weight every 24 h, administered in four to five doses [3, 31, 32]. It is important to note that diarrhea is one of the effects of oral phosphate overdose, which can contribute to further electrolyte imbalance. Our patient received higher doses (62 mg Pi/kg body weight over 24 h) with good tolerance.

Intravenous administration of phosphate solution should be used for critically ill patients and those who are nil by mouth. Both potassium and sodium phosphates are available. These should be administered together with sodium chloride or glucose solutions, as low-soluble salt would precipitate in solutions containing calcium. High dose or rapid phosphate infusion may lead to hypophosphatemia, hypotension, hypocalcaemia with tetanus, renal failure and ECG abnormalities. Most of the data about dosage has come from research on adults, thus each case must be approached with caution. A dosage of 0.08 mmol/kg body weight every 6 h is recommended for short-term uncomplicated hypophosphatemia, whereas a dosage of 0.16 mmol/kg body weight is advised for long-term hypophosphatemia of complex origin. Some manufacturers recommend a maximum single phosphate dosage of 0.24 mmol/kg body weight, although some authors have reported the use of doses of 0.4–0.5 mmol/kg body weight every 6 h in specific conditions, up to a maximum dose of 50 mmol. Doses should be individually adjusted to the patient’s clinical condition, hypophosphatemia severity, and their reaction to phosphate infusion [3, 6, 32, 33].

## Conclusions

It is recommended that blood calcium and phosphate levels be carefully monitored in DKA patients in a serious condition. The blood phosphate level decreases after the initiation of DKA treatment, which is potentiated by insulin. Clinical symptoms usually occur at a blood phosphate level lower than 0.32 mmol/L, although the intracellular phosphate level also plays a crucial role. Hypophosphatemia commonly appears on the second day of treatment, thus it is vital to monitor ECG. Severe hypophosphatemia with clinical symptoms requires oral or intravenous supplementation of phosphate. So far, there is no evidence of any benefits of routine administration of phosphate in patients with DKA. Patients with DKA who are at risk for hypophosphatemia include those with low blood pH, extremely high blood glucose level and very low BE, nil by mouth patients who have received intravenous insulin for longer than 24 h, cachectic or malnourished patients, and those with a vitamin D deficiency.

## Abbreviations

2,3-DPG: 2,3-Diphosphoglycerate
ATP: Adenosine triphosphate
BE: Base excess
DKA: Diabetic ketoacidosis
ECG: Electrocardiography
LBBB: Left bundle branch block
PTH: Parathormone
PTHrP: Parathyroid hormone-related protein
PVC: Premature Ventricular Contractions
SVT: Supraventricular tachycardia
